# Dry Eye Disease: A Review of Epidemiology in Taiwan, and its Clinical Treatment and Merits

**DOI:** 10.3390/jcm8081227

**Published:** 2019-08-15

**Authors:** Yu-Kai Kuo, I-Chan Lin, Li-Nien Chien, Tzu-Yu Lin, Ying-Ting How, Ko-Hua Chen, Gregory J. Dusting, Ching-Li Tseng

**Affiliations:** 1 School of Medicine, College of Medicine, Taipei Medical University, Taipei 11031, Taiwan; 2 Department of Ophthalmology, Shuang Ho Hospital, Taipei Medical University, New Taipei City 23561, Taiwan; 3 Department of Ophthalmology, School of Medicine, College of Medicine, Taipei Medical University, Taipei 11031, Taiwan; 4 School of Health Care Administration, College of Management, , Taipei Medical University, Taipei 11031, Taiwan; 5 Health and Clinical Data Research Center, College of Public Health, Taipei Medical University, Taipei 11031, Taiwan; 6 Graduate Institute of Biomedical Materials & Tissue Engineering, College of Biomedical Engineering, Taipei Medical University, Taipei 11031, Taiwan; 7 Department of Ophthalmology, Taipei Veterans General Hospital, Taipei 11217, Taiwan; 8 Centre for Eye Research Australia, Royal Victorian Eye and Ear Hospital, East Melbourne, VIC 3002, Australia; 9 Ophthalmology, Department of Surgery, University of Melbourne, East Melbourne, VIC 3002, Australia; 10 Institute of International PhD Program in Biomedical Engineering, College of Biomedical Engineering, Taipei Medical University, Taipei 11031, Taiwan; 11 Research Center of Biomedical Device, College of Biomedical Engineering, Taipei Medical University, Taipei 11031, Taiwan; 12 International PhD Program in Cell Therapy and Regenerative Medicine, College of Medicine, Taipei Medical University, Taipei 11031, Taiwan

**Keywords:** dry eye disease (DED), prevalence, risk factor, subtype, DED treatment, nanomedicine

## Abstract

Dry eye disease (DED) has become common on a global scale in recent years. There is a wide prevalence of DED in different countries based on various ethnicities and environment. DED is a multifactorial ocular disorder. In addition to advanced age and gender, such factors as living at high altitude, smoking, pterygium, prolonged use of consumer electronics or overingesting of caffeine or multivitamins are considered to be the major risk factors of DED. We report the DED epidemiology in Taiwan firstly in this article. According to the pathophysiological factors and changes inthe composition of the tear film in DED, it can be categorized into several subtypes, including lipid anomaly dry eye, aqueous tear deficiency, allergic and toxic dry eye among others. Each subtype has its own cause and disease management; therefore, it is important for ophthalmologists to identify the type through literature review and investigation. The management of DED, relies not only on traditional medications such as artificial tears, gels and ointments, but also newer treatment options such as acupuncture, SYL1001, and nanomedicine therapy. We also conducted a comprehensive literature review including common subtypes and treatment of DED. Clearly, more clinical trials are needed to assess the efficacy and safety of the various treatments and common subtypes of DED.

## 1. Introduction

Dry eye disease (DED) is a generic ocular disorder extremely common all over the world. One fourth of the patients visiting an ophthalmic clinic complain of suffering from the symptoms of dry eye. In recent years, DED has become an inevitable public health problem [1]. In 2017, the Tear Film and Ocular Surface Society (TFOS) Dry Eye Workshop (DEWS) II reviewed the definition of dry eye disease previously presented in 2007 [2]. Compared to the 2007 definition, which combined the roles of tear hyperosmolarity, ocular surface inflammation and DED associated classification based on etiology, mechanism and severity, a major change in the new definition is “a loss of homeostasis” [3]. This was highlighted by the subcommittee as the unifying characteristic that describes the fundamental process in the development of DED. The updated definition is “Dry eye disease is a multifactorial disease of the ocular surface characterized by a loss of homeostasis of the tear film, and accompanied by ocular symptoms, in which tear film hyperosmolarity and instability, ocular surface inflammation and damage, and neurosensory abnormalities play etiological roles [2].

Based on the clinical use and literature review, several examinational tools that are available to diagnose and classify DED are presented in Table 1. These assessments are important to distinguish the various subtypes of DED including infections and allergies, which could present very similar clinical symptoms, but require different managements. An incorrect diagnosis or prescription of drugs may actually end up worsening the DED [4]. Regarding these assessment tools, a cross-sectional study was conducted to survey the concordance between patient and clinician assessment of DED severity and treatment response in Taiwan. Individuals enrolled in the trial were over 20 years of age with suspected DED defined by at least one positive Schirmer test (less than 10 mm per 5 min) in at least 1 eye within the past 12 months. For assessing the severity of DED, clinicians used the Dry Eye Workshop severity (DEWS) grading scheme, and patients completed the Ocular Surface Disease Index (OSDI) questionnaire. Based on the slit-lamp examination, assessment of meibomian gland dysfunction, ocular surface fluorescence staining (Oxford scheme), and tear film break-up time, dry eye severity was graded from level 1 to level 4, with higher levels indicating a more severe DED condition. The Oxford scheme was conducted to estimate surface damage in dry eye, which was divided into six groups through the relationship between punctate dots and series panels by staining photograph [5]. And the OSDI questionnaire consists of twelve items designed to assess ocular symptoms, vision-related function, and environmental triggers. Based on the OSDI score, the ocular surface was defined as normal (0–12), mild (13–22), moderate (23–32), or severe (33–100) [6]. Besides, in order to evaluate the treatment response, patients completed the Subject Global Assessment scale, and clinicians independently assessed patients using the Clinical Global Impression scale. According to their analysis, the result showed that there was low agreement between clinician and patient assessments in terms of disease severity and treatment response. Clinicians may underestimate DED severity and the persistence of dry eye symptoms after treatment with artificial tears among different subgroups based on gender and age [7]. Hence, the need for further defining DED severity, as well as the routine use of a validated disease-specific questionnaire is recommended to ensure better treatment choices. A comprehensive history of DED is also necessary, including time, place and diurnal variation of symptoms and working pressure. Identifying systemic disease (especially collagen vascular disease, Graves’ disease and etc.) and medical history is also essential for ophthalmologists [8].

The economic burden of DED, including direct costs from doctor visits, medications, procedures and indirect costs from decreased productivity, reduced quality of life, and general impairment in functioning have affected patients significantly [21,22]. An economic analysis reveals that the average annual cost of managing a patient with DED is $783 in the United States. When adjusted to the prevalence of DED nationwide, the overall burden of DED for the US healthcare system would be around $3.84 billion [23]. Therefore, the impact of DED presents important economic challenges not only to patients, but also physicians and health care delivery organizations [24].

## 2. Epidemiology

### 2.1. Prevalence and Incidence in Taiwan

The prevalence of dry eye disease varies globally in different places, ranging from 7% in the United States to over 30% in Taiwan, Japan and Korea [25,26,27]. It is believed that this difference in the prevalence of DED between Asian and Western populations is based on ethnic variability [28]. In Taiwan, a population-based survey of eye disease showed that 33.7% of the elderly were symptomatic, defined as reporting one or more dry-eye symptoms [29]. According to a cohort study through the National Health Insurance Research Database (NHIRD) from 2000 to 2008 in Taiwan, the incidence rates of developing Sjogren’s syndrome were 4.8% for the group of dry eye disease and 1.5% for the comparison group. In addition, patients with DED presented for Sjogren’s syndrome were diagnosed 3.88 years earlier than the patients in the comparison group [30].

**Figure 1:**
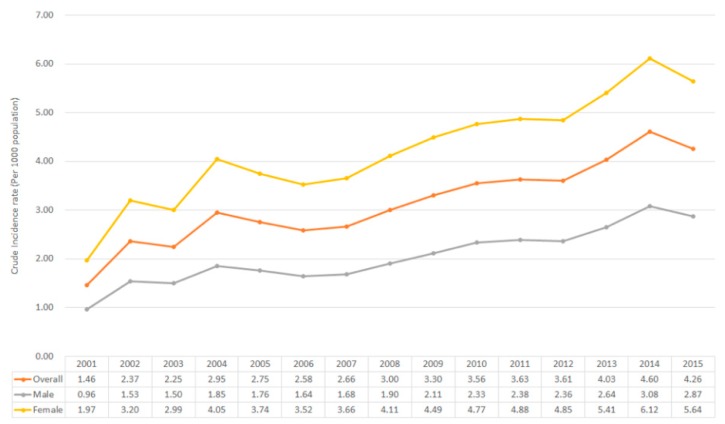
Crude incidence (per 1000 population) of DED by year and gender.

**Figure 2:**
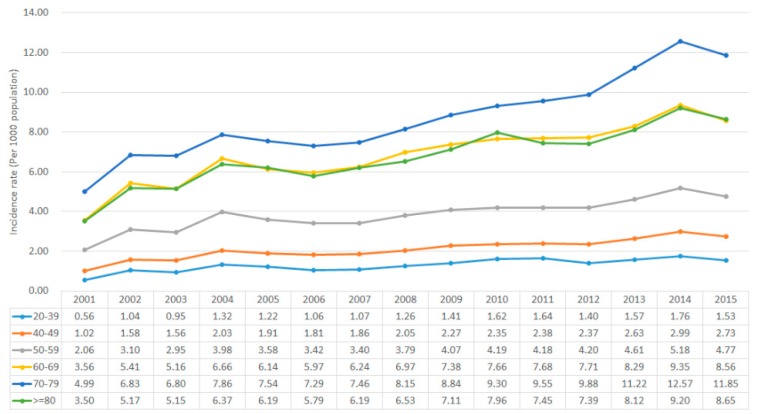
Age-specific incidence related to DED by year.

In order to determine the incidence of DED in Taiwan, we conducted a population-based study through the data from NHIRD since 2001–2015. The International Classification of Diseases, Ninth Revision, Clinical Modification (ICD-9-CM) contains a list of codes corresponding to diagnoses and procedures recorded in conjunction with hospital care in the United States. The main purpose of ICD-9-CM was used for diagnostic, billing and reporting purposes. In addition, other information including symptoms, patient complaints, causes of injury, and mental disorders were also classified and codified in this system. Via ICD-9-CM, we could provide better consistency among physicians in recording patient symptoms and diagnoses for the purposes of enhancing clinical research in different countries and areas. The study group comprised all patients who sought ambulatory care with two principal diagnoses of dry eye disease (ICD-9-CM code of 375.15 referred to tear film insufficiency, unspecified and 370.33 referred to keratoconjunctivitis sicca, not specified as Sjogren’s) but without a history of DED from 2001 to 2015 (*n* = 3,019,377). To ensure the validity of disease diagnoses, we selected only those patients who had at least two consensus DED diagnoses within half a year and had been prescribed eye lubricants with S01XA18 or A01XA20 during this period (*n* = 764,611). The S01XA18 and A01XA20 are the two categorized in the Anatomical Therapeutic Chemical (ATC) classification system of the Ophthalmological ATC code S01. The S01XA18 represents ophthalmological agents containing cyclosporine, and A01XA20 is the code showing artificial tears/eye lubricants related agents. We excluded patients under 20 years of age (*n* = 25,869) to limit the study sample to the adult population. In addition, we excluded patients who had been diagnosed with Sjogren’s syndrome during the whole study period (ICD-9-CM code 710.2, sicca syndrome) (*n* = 146,085). In total, 764,611 patients with DED were eligible for inclusion in this study. Between 2001 to 2015, there were 764,611 newly diagnosed patients with DED. The analysis of incidence, crude incidence and age-specific incidence are common tools for scientists to observe trends in a population-based study. Crude incidence is simple and straightforward, it is calculated by dividing the total number of cases in a given time period by the total number of persons in the population category of interest. However, it does not reflect a fair comparison accurately if there is an unequal age distribution in each group. Hence, age-specific incidence offers helpful solutions. Here, we show the annual incidence rate which was equaled to the total number of new DED cases diagnosed in a year divided by the at-risk population and multiplied by 1000. The result regarding the annual crude incidence rate shows an increasing trend from 1.46 per 1000 population in 2001 to 4.26 per 1000 population in 2015 (Figure 1). Besides, the result also recognized that the incidences of DED in women were continuously higher than the number of incidents in male patients while the incident rate ratios (female: male) were relatively constant over a 15-year study period. In Figure 2, it shows annual incidence rates by age group. The incidence rates of DED generally increased with age, but decreased in the population of age 80 and older, which might reflect lower rates of disease awareness and/or incomplete detection. Our finding was consistent with many studies [25,31,32].

### 2.2. Risk Factors

There are several risk factors related to dry eye disease, particularly in the elderly, postmenopausal women and those suffering from autoimmune diseases [33,34]. The classification according to the National Eye Institute (NEI) divides dry eye disease into the aqueous-deficient type and the evaporative type [35]. Under each basic division, there are many different subtypes [36]. It is thus very important to understand the pathophysiology of each subtype because it will affect the choice of treatment. The present study, therefore, incorporates new insights into DED, especially the common clinical subtypes and new treatment approaches.

In addition to Sjogren’s syndrome, a higher risk of chronic fatigue syndrome, and oral cavity cancer was also found in the patients with DED [37,38]. Furthermore, environmental factors such as carbon monoxide (CO), nitrogen dioxide (NO₂), and temperature were positively associated with dry eye disease, while relative humidity was negatively related [39]. In Taiwan, several studies revealed that osteoporosis, asthma, fibromyalgia, gout, presbyopia and the increased use of glaucoma medications have increased the risk of developing dry eye disease [40,41,42,43,44,45]. In terms of autoimmune rheumatic diseases, one population-based cohort study also shows that patients with palindromic rheumatism had an increased risk of developing rheumatoid arthritis, systemic lupus erythematosus, systemic sclerosis, polymyositis and dermatomyositis [46].

Moreover, other risk factors such as high altitude, pterygium, smoking, excessive ingestion of multivitamins and caffeine, and poor quality of life, are also reported to be associated with DED [47,48,49]. On the other hand, a systematic review from China categorized DED into two diagnostic groups: “DED by symptoms and signs” and “DED by symptoms”. DED by symptoms and signs was defined as a positive symptom with at least one positive clinical sign tested by tear film breakup time ≤10 s, Schirmer’s I test ≤10 s or fluorescein staining ≥1, whereas DED by symptoms only relied on the presence of self-reported positive symptoms. These results support that, despite advanced age, female sex and larger latitude were significant risk factors for dry eye based on symptoms and signs; only advanced age was positively associated with an increased prevalence of DED by symptoms [50].

## 3. Pathophysiology

**Figure 3:**
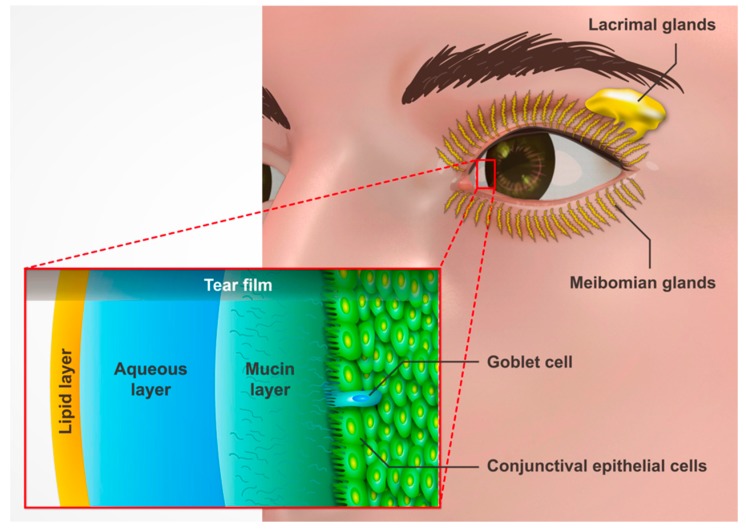
Structures involved in the production of tear film.

The tear film is composed of three main layers which are the outer lipid, the middle aqueous and the inner mucus layers. (Figure 3) [51]. Each layer has its different composition and function. The lipid layer is the outermost layer which is secreted by the Meibomian glands and the glands of Zeis. In the past, its function was recognized to decrease the evaporation of the aqueous layer beneath it [52]. However, some recent research demonstrated that the lipid layer may not inhibit the rate of evaporation [53]. One study showed that it is unlikely that hydroxyl lipids secreted from the Meibomian glands can be used to inhibit the rate of evaporation among reservoirs exposed to the lachrymatory factor in the vapor of freshly cut onions for three minute intervals [54]. There are actually only a few studies that focus on the single main function of the lipid layer. According to most of studies, other functions of the lipid layer include providing a low surface tension for tear film, viscoelastic films capable of opposing dilation of the air–tear interface, and spreading the tears between blinks [55,56,57,58,59]. The middle, aqueous layer is the thickest layer. It is produced by the acinar cells from the glands of upper lids and the accessory tear glands, which keeps the eye moist and helps in the removal of debris or foreign particles [60]. The innermost layer is the mucus layer, which is secreted by specialized goblet cells in the conjunctival epithelium [61]. It helps the overlying aqueous layer to spread evenly over the cornea [62]. The instability of the tear film is one of the most common causes of dry eye. Besides, dry eye is recognized as a result of disruption of the lacrimal functional unit, which is composed of lacrimal glands, cornea, conjunctiva, eyelids, Meibomian glands, ocular nerves, and goblet cells [63]. Some studies also revealed that inflammation is another core mechanism of DED, which is mediated by lymphocytes within the conjunctiva [64,65,66,67]. T cell infiltration and upregulation of CD3, CD4, and CD8 as well as lymphocyte activation markers CD11a and HLA-DR are found in conjunctival biopsy specimens among Sjogren syndrome-related and non-Sjogren syndrome-related DED [68]. The chronicity of the disease suggests that the dysregulation of immune mechanisms leads to a cycle of continued inflammation, accompanied by alterations in both innate and adaptive immune responses [69].

## 4. Different Subtypes of Dry Eye Disease

The aqueous deficient and the evaporative types of DED can be sub-divided into several subtypes resulting from anomalies in gland, lid or blinking function. These include lipid anomaly dry eye (LADE), allergic and toxic dry eye (ADE), cicatricial condition, autoimmune condition, lid surfacing anomalies (LSADE), etc., [36].

### 4.1. Lipid Anomaly Dry Eye (LADE)

Lipid anomaly dry eye (LADE) is often associated with dysfunction of the Meibomian gland, which is the leading cause of evaporative dry eye [70,71]. Increased tear evaporation due to a compromised lipid layer is one of the common causes for hyperosmolarity change of the tear film [72]. It is also recognized as the most prevalent subtype of dry eye [72,73]. There were several treatments to improve the quality of the Meibomian gland in the past, including warm compresses and improved eyelid hygiene, as well as antibiotic and anti-inflammatory agents. To overcome the shortcomings of the above, there are more options for the treatment of dysfunction of Meibomian gland currently, such as intraductal Meibomian gland probing, emulsion eye drops containing lipids, thermal pulsation system, and intense pulsed light therapy [74,75,76,77,78]. One systemic review also confirmed that a single 12-min vectored thermal pulsation was more efficient than traditional warm compression for DED in both objective or subjective measurements. In this review, the objective measurement included gland function, tear break up time (TBUT), the Schirmer test, tear osmolarity, lipid layer thickness, whereas the subjective measurement was based on the Ocular Surface Disease Index [79]. Different heated devices have been developed to warm the eyelid as a therapy for Meibomian gland dysfunction treatment, Borchman D reveals that the safety and tolerable temperature for heating the eyelide not to cause the disorder of Meibomian gland should be between 30–34 °C for warm treatment [80].

### 4.2. Aqueous Tear Deficiency (ATD)

Aqueous tear deficiency (ATD) is a multifactor cause of dry eye disease, which is related to autoimmune disease such as Sjogren’s syndrome. Though both Sjogren’s syndrome and non-Sjogren’s syndrome present with similar sicca symptoms of dry eyes and dry mouth, there are several differences such as the severity of symptoms, Ocular Surface Disease Index (OSDI), sialoscintigraphy, and serum a-fording, Ro and La for discrimination [81,82,83]. Sjogren syndrome is an autoimmune disease associated with lacrimal and salivary gland lymphocytic infiltration [84]. These glands show the infiltration of T cell, B cell, dendritic, and natural killer cells, while antigen-presenting cells have also been reported in heavy infiltrates [85]. Due to the unique pathophysiology, compared to the treatment of other subtypes, the treatment of Sjogren syndrome-related dry eye further emphasizes anti-inflammatory and immune regulatory agents, such as essential fatty acids, autologous serum, secretagogues, and corticosteroid [86].

Cicatricial pemphigoid is another autoimmune disease under the classification of aqueous tear deficiency. It often causes dry eye due to lacrimal gland dysfunction and conjunctival cauterization [87]. Besides, loss of vision usually occurs during the progression of the disease. Cicatricial pemphigoid-related dry eye often affects the bilateral eye and is more common in females, with most cases occurring between 30–90 years. Similar to Sjogren syndrome, the control of conjunctival inflammation is mandatory to prevent disease progression and requires systemic immunosuppressive therapy.

In recent years, several medical treatments have been introduced, including steroid, infliximab, daclizumab, methotrexate and intravenous immunoglobulin therapy, that have been applied in clinical care [88,89,90,91]. However, the effect of subconjunctival mitomycin remains controversial. Some studies have given it recognition as being efficacious in controlling long-term conjunctival inflammation, while others have not [92,93]. According to the previous study conducted by Juri MC et al., methotrexate and azathioprine were effective at relieving dry eye with low toxicity. Dapsone, conversely, was only useful in mild cases and showed frequent adverse effects, and IV Ig was found effective against refractory cases, which is consistent with the results of another retrospective study [94,95]. Other management for cicatricial pemphigoid includes plastic surgery for lid and lash malposition, tetracyclines and lid hygiene for the blepharitis [96].

Other than DED, these autoimmune diseases are usually accompanied by other symptoms. Hence, the treatment cannot be the lack of control of symptoms over another organ. Overall, disease management must be integrated with other conditions such as ocular surface disease and inflammation [87]. For dry eye, lubricants without preservatives is suggested to avoid any toxic reaction [97].

### 4.3. Allergic and Toxic Dry Eye (ADE)

Dry eye due to allergy or toxin is usually associated with inflammatory processes which could destroy the glycocalyx and mucin layers. For some patients presenting only with decreased tears, break-up time related to decreased goblet cell density, allergic conjunctivitis is usually the main cause of ADE [97,98,99]. Some studies also indicated that most patients with “itchy eyes” consistent with allergic conjunctivitis also have dry eyes and redness, even in the pediatric population [100,101].

### 4.4. Lid Surfacing or Blinking Anomalies (LSADE)

Blink rate has been recognized to have a strong association with dry eye for a long time because of its ability to determine tear film stability. Usually, reduced and incomplete blinking along with an increased tear film break-up during normal visual tasks would result in ocular discomfort symptoms. Previous studies have shown that healthy people blink significantly less than patients diagnosed with dry eye during visual functional tasks, such as watching TV, using a computer and reading [102,103]. In addition to blink rate, blink patterns, total lid-contact time and interblink interval (IBI) are also evaluated while examining patients with dry eye [104]. Interblink interval (IBI) was defined as the duration between the onset of eyeblink response and the onset of the next eyeblink response in a time series, total audience eyeblinks were recorded with a particular time interval [105]. One study showed that, compared to normal patients, IBI was significantly shorter for dry eye patients performing a visual task [106]. The results from another study also suggested that the total contact time was seven times higher in patients with dry eye than normal patients [107]. Furthermore, no confirmed significant correlations between blink speed and symptoms or tear film stability have been found among dry-eye patients [108].

### 4.5. Marginal Dry Eye

The classification of marginal dry eye is often based according to individuals whose tear function is adequate only in favorable conditions. However, in provocative circumstances such as air conditioning, central heating, usage of dehydrating medications, alcohol consumption, and wearing of contact lenses, their tear function would be affected greatly [109].

### 4.6. Cicatricial Condition

Cicatricial condition often presents as dryness of the conjunctiva and the cornea, which increases the risk of infection in patients [110]. Xerophthalmia and trachoma are two of the common causes of dry eye related to cicatricial condition in developing countries. Patients with xerophthamia generally live in urban areas and are not usually able to consume the recommended and sufficient levels of Vitamin A. Such patients are often prescribed with topical retinoic acid [111,112]. Various animal tests have revealed that tretinoin (all-trans-retinoic acid) is very efficient in reversing xerophthalmic changes compared to retinol [113].

Chlamydia trachomatis is another factor that contributes to cicatricial condition. Higher prevalence of trachoma in urban areas are due to poor hygiene, dusty environments and low socio-economical conditions [114]. This species not only affects the superficial epithelium with follicles and papillary hyperplasia, but trachoma also destroys the functioning of lids, lacrimal and goblet glands leading to symptoms of dry eye [115]. Antibiotics are considered as an effective method of controlling Chlamydia trachomatis. A series of randomized clinical trials (RCT) conducted on patients with trachoma indicated that a single dose of oral azithromycin is far more effective that a topical ointment containing tetracycline [116].

## 5. Treatment and New Therapeutic Agents for DED

According to the causes of each DED subtype, there are several treatments for dry eye disease or tear film dysfunction, such as artificial tears, punctal plugs, warm compression, prescription medicine, topical ophthalmic steroid, and mucin secretagogue among others. Other forms of therapy were also designed to control specific mechanisms, including topical cyclosporine A, autologous serum, and sodium hyaluronate drops, which suppress underlying inflammation, provide growth factors, and prevent the onset of squamous metaplasia in ocular surface epithelium [1,117,118,119].

### 5.1. Artificial Tear

Artificial tear has been the mainstay of therapy for all severity grades of dry eye for several years. It provides more tear stability, more contrast sensitivity and less ocular surface stress. Hence, many studies have reviewed the efficacy, safety and tolerability of artificial tear from each company. A randomized, single masked crossover trial showed that osmolarity balanced artificial tears were the preferred treatment in patients with low tear volume and liposomal spray for patients with lipid layer deficiency [120]. From baseline within 30 days with use of 0.5% carboxymethylcellulose sodium (CMC)/0.9% Glycerin (GLY) eye drops or 0.5% CMC artificial tears, dry eye signs and symptoms based on Schirmer Test, tear break up time (TBUT), corneal and conjunctival staining and OSDI are confirmed to have a significant improvement [121]. Similar findings were also found in another study which evaluated 1.0% CMC/0.9% GLY and 1.0% CMC eye drops for patients with moderate to severe dry eye [122]. Administration of seawater is thought to be more effective than treatment with carmellose artificial tears in reducing pro-inflammatory molecules, such as IL-1 beta and IL-6, in tears among patients with DED [123].

### 5.2. Artificial Solution without/with Anti-Inflammatory Drugs

Artificial solutions are lubricant agents. They are easy to apply and hence the first line of treatment for eye diseases. Hyaluronic acid (HA) is one of the topical agents used in artificial solutions to treat dry eye disease. A clinical report involving 86 participants with DED found that sodium hyaluronate artificial solution has a beneficial effect in reducing ocular surface damage [124]. Moreover, 0.3% HA has been proven more effective than 0.1% HA and 0.18% HA to treat dry eye syndrome because it is able to improve tear film instability, ocular surface staining and irregularity, increase the number of conjunctival goblet cells and decrease corneal epithelial apoptosis [125]. Tacrolimus is a steroid-sparing anti-inflammatory agent used to replace steroid drugs that are potentially associated with side effects after long term use. A report of clinical outcome shows that 0.03% tacrolimus eye drops (olive oil + 0.03% tacrolimus) improves tear stability and ocular surface status in patients with dry eye after 90 days. To improve the effect of artificial solution in treating DED, recent studies combine more than one drugs in artificial solution. The efficacy of an artificial solution containing epigallocatechin gallate (EGCG) and HA was examined in rabbits with DED and revealed improvement of tear secretion, fewer apoptotic cells in cornea, lower production of inflammatory cytokines such as IL-6, IL-8, and TNF-α [126]. An artificial solution containing omega-3 EFA and HA also demonstrated successful treatment for DED by improving corneal irregularity, corneal epithelial barrier disruption, as well as decreasing inflammatory cytokines and oxidative stress markers on the ocular surface [4,127].

### 5.3. Cyclosporine A

Cyclosporine A possesses a novel amino acid peptide with N-methylated amino acids resulting in a cyclic structure [128]. It has long played an important role in the field of the treatment of various immune-mediated disorders, such as psoariasis, and organ transplantation [129,130,131]. Topical cyclosporine A (tCSA) seems to be a promising treatment for DED since it is the first agent focused on the pathogenesis of this disease [132,133,134,135]. It works to restore the ocular surface, allowing increased production of tears by inhibiting the T-cell activation pathway [64,136]. tCSA reduces the symptoms of dry eye by modulating the cell-mediated inflammatory cascade. In terms of the dosage of tCSA for DED, it is different from the treatment of immunosuppression dosage based on the calculation of bodyweight in terms of mg/kg [137].

The most common dosage of tCSA used in DED is the Restasis with 0.05% tCSA [138]. One study showed a significant improvement observed after an initial treatment with tCSA and a 10-year follow-up [139]. Another study reported patients showing improvement after only 30 days of therapy with tCSA. This result suggests that tCSA provides a faster onset of symptomatic relief [140]. One retrospective cohort study suggests that patients with severe dry eye may require more frequent dosing of tCSA 0.05% than twice daily [141]. Conversely, another study evaluated the effect for the decrease of the frequency of tCSA from twice daily to once daily among patients with dry eye. Those patients with dry eye had been controlled with tCSA twice daily for at least 1 year. These findings supported that decreasing tCSA administration to once daily still resulted in the suppression of DED [142]. An animal study conducted in 2015 compared the efficacy of 1% cyclosporine eye drops in olive oil or linseed oil for the treatment of experimentally-induced keratoconjunctivitis sicca (KCS) rabbits. The results support that cyclosporine diluted in both olive oil or linseed oil was effective in the treatment of KCS, although it had better efficacy in linseed oil [143]. These findings may give birth to the creation of novel topical ophthalmic formulations in future. In addition, goblet cell density, corneal sensitivity, and tear meniscus height and volume were found to improve with tCSA [144,145,146,147].

### 5.4. Autologous Serum

Several studies have pointed out the great effectiveness of autologous serum for DED [148,149,150]. Autologous serum is potentially advantageous to treat many dry eye-related ocular surface disorders, such as Sjogren’s syndrome, graft-versus-host disease, Stevens–Johnson syndrome, and ocular cicatricial pemphigoid. Human serum contains different biochemical components such as epidermal growth factor, vitamin A, transforming growth factor-β, fibronectin, and cytokines [151]. These substances are normally found in tears, which lubricate the ocular surface and maintain a healthy corneal and conjunctival epithelium. One systemic review evaluated the efficacy and safety of autologous serum compared to artificial tears for treating dry eye. The result showed that in some trials, autologous serum may provide improvement in participant-reported symptoms compared to traditional artificial tears after two weeks of treatment. However, larger and higher-quality RCTs are warranted to assess different severities of dry eye in terms of the use of autologous serum [152].

### 5.5. Punctal Plug

Punctal plug is another common therapy for DED. Lacrimal occlusion with plugs prolongs the effects of lubricants and preserves natural tears. They are relatively contraindicated in DED patients and coexisting inflammation [153]. A systematic review assesses the effectiveness of punctal plugs for managing dry eye. Results show that compared to artificial tears, punctal plug participants have more symptomatic improvement after a period of three months. Besides, punctal plug placement with acrylic, silicone, cyclosporine or pilocarpine, show no statistically significant difference in symptomatic improvement at 2–12 months [154]. Although punctal plugs are believed to be relatively safe, some complications were also found in patients with DED after their use, including extrusion, granulation, pyogenic granuloma, and canaliculitis [155,156,157,158]. Reviews and findings suggest that punctal plugs have a higher rate of epiphora and plug loss than intracanalicular plugs, whereas permanent intracanalicular plugs have a higher association with canaliculitis and pyogenic granulomas [159].

### 5.6. Warm Compression

Warm compression has long been recommended as a treatment for Meibomian gland dysfunction (MGD) [160,161]. The tear-film lipid layer thickness (TFLLT) was found to increase more than 80% following 5 min after initial warm, moist compress therapy, whereas an additional 20% following 15 min [162]. Besides, compared to warm towel compressions which required reheating to maintain the same temperature, a hydrating Mask may be a more convenient treatment alternative for MGD with its once-only heating advantage [163]. One study also confirmed that a warm compress containing menthol is a potential novel treatment for DED because menthol has been recognized to stimulate lacrimation via activation of cold-sensitive primary afferent neurons in the cornea [164]. Elevated eyelid temperature delivers more meibomian oil to the eyelid [165], and it is a way to ameliorate dry eye symptoms by warm treatment. However, too high a temperature should be avoided as heat could cause injury to the eyelid skin, and also contribute to presbyopia and cataract [80,166]. Borchman D proved that heating the eyelid above the phase transition temperature (Tt) of meibum, which is 30–34 °C, can slightly increase the disorder of meibum lipid to ameliorate dry eye symptoms [80]. At higher tehperature (>40 °C), the heat could cause Meibomian gland dysfunction reach up to 90%. The discomfort and safety issues should be considered.

### 5.7. Mucin Secretagogue

Mucin secretagogue is confirmed to stimulate glycoprotein secretion in human ocular tissue at submicromolar concentrations, and provide therapeutic benefit to the injured cornea in the dry eye condition [167]. In 2002, Gamache DA. conducted an animal model to demonstrate that mucin secretagogue 15(S)-HETE could stimulate ocular mucin secretion in vitro and protect the cornea in a rabbit model of desiccation-induced injury. The results supported that the ocular mucin secretagogue 15(S)-HETE may have therapeutic utility in dry eye patients through alleviating corneal injury and restoring corneal integrity [168]. Rebamipide, a novel quinolinone derivative synthesized, was used in treating gastric ulcers and lesions related to gastritis due to its ability to increase gastric mucin [169]. Now it has become dry eye medication following the investigation of its effect on the ocular surface mucin [170,171]. One prospective study was carried out to evaluate the efficacy of 2% rebamipide ophthalmic solution for DED. In the study, there are forty eyes of patients having signs and symptoms of dry eye based on dry eye-related symptom score, TBUT, tear meniscus height, fluorescein ocular surface staining score (FOSS), and the Schirmer’s test. The results suggested that 2% rebamipide ophthalmic solution provided relief in the symptoms of patients with DED. In addition, it also prevented further ocular surface damage and helped stabilize the tear film [172,173]. Another study also figured out that 2% rebamipide may be more effective than 1% rebamipide [174]. Additionally, the most important secreted mucin on the ocular surface is MUC5AC, which is secreted by the conjunctival goblet cells [175]. The mechanism of the stimulation of mucin secretion or the increase the number of conjunctival goblet cells have been designed to be novel drugs for DED, such as diquafosol, a P2Y2 receptor agonist approved in Japan [176,177]. There is also a randomized clinical trial investigated the dose-dependent efficacy and safety of diquafosol. The result showed that both 1% and 3% diquafosol ophthalmic solutions are effective and safe for the treatment of DED [178].

### 5.8. Anti-Inflammatory Agents (Steroid and Non-Steroid)

Corticosteroids, such as dexamethasone, loteprednol etabonate, prednisolone, and fluorometholone, are effective steroid anti-inflammatory agents widely used in curing dry eye syndrome. They can suppress MMP-9 and inflammatory cytokine expression, MAPK activation in the cornea epithelium [179]. However, they cause numerous side effects, notably cataract and intraocular pressure evaluation, and increase the risk of infection in long term use [180,181]. Treatment by applying steroidal anti-inflammatory agents has a high potential for inducing many sides effects and hence, steroid-sparing anti-inflammatory agents have also been investigated.

For patients suffering from severe keratoconjunctivitis sicca with eye irritation despite maximum aqueous enhancement therapies, topical ophthalmic steroid is an effective option [181,182]. However, it is important to follow up on steroid-related complications such as increased intraocular pressure and cataract formation, especially if patients were steroid-responders [183]. One study reviewed the efficacy and side effects of two different topical corticosteroid schemes (pulse vs. tapered) for DED. In their method, patients were treated with pulse (q.i.d. for 2 weeks) or tapered (t.i.d. for 1 week, b.i.d. for 2 weeks, q.d. for 4 weeks, every other day for 8 weeks) therapy. The result revealed that topical loteprednol etabonate 0.5% is a safe and effective treatment for patients with moderate to severe dry eye. In addition, the tapered small dose approach seems to provide a better control of symptoms, requiring a lower rate of retreatments [184]. In 2018, S Singla conducted one prospective clinical trial and found that combination therapy with topical loteprednol 0.5% and topical cyclosporine 0.05% is significantly better than topical cyclosporine 0.05% alone, for alleviating symptoms and signs based on OSDI, TBUT, corneal fluorescein, and lissamine green staining scores in moderate dry eye patients [185].

### 5.9. Other Potential Agents

Recently, many kinds of treatments for DED have been proposed, such as Meibomian gland squeezer, polyunsaturated fatty acids, omega-3 fatty acids, perfluorohexyloctane, chitosan-N-acetylcysteine (C-NAC) and retinol palmitate [186,187,188,189,190,191]. In terms of omega fatty acids, studies demonstrated that ω-3 and ω-6 fatty acids can act directly on human Meibomian gland epithelial cells to influence the quality and quantity of intracellular lipids [192]. Dietary supplementation with a combination of ω-3 and antioxidants was found to have a significant beneficial effect on health-related quality of life in patients with MGD [193]. One double-blinded randomized clinical trial showed that among patients with dry eye, there is no significant better outcomes between omega-3 fatty acids and placebo [194]. As for C-NAC, an animal study surveyed its effect on a botulinum toxin B-induced dry eye mouse model. The results suggested that C-NAC may impart some protective ocular surface properties [195]. Another animal clinical trial indicated that C-NAC had a positive impact on corneal wound healing [196]. Retinol palmitate is known to regulate the proliferation and differentiation of corneal epithelial cells and preserved conjunctival goblet cells. Its mechanism is related to the inhibition of VEGF-A and the activation of thrombospondin 2 [197]. Hence, it is widely used in the treatment of DED [198]. For patients with dry eye failing to respond to the conventional therapy with artificial tears or cornea-protective drugs, one study found an increase in goblet cells, a decrease in keratinized cells and an increase in non-keratinized cells after treatment with retinol palmitate [199]. These findings gave retinol palmitate recognition in the treatment of DED. According to one clinical trial from Taiwan published in 2019, the therapeutic effects of a pigment epithelium-derived factor (PEDF) peptide was found to reverse mouse age-related meibomian gland atrophy. The mechanism behind it was confirmed to be related to promoting meibomian gland acinar basal cell proliferation [200].

Acupuncture has become another complementary and alternative treatment especially for patients unsatisfied with conventional treatments. However, the efficacy of acupuncture remains controversial [201,202,203]. In addition, another therapy called SYL1001, which is a novel short interfering RNA (siRNA) targets the transient receptor potential cation channel subfamily V member 1 (TRPV1), known as a capsaicin receptor, which mediates transmission of painful stimuli and inflammatory responses [204,205].

## 6. A New Trend for Applying Nanomedicine in DED Treatment

The application of nanoparticles in ocular diseases allows targeted delivery, slow release, and enhanced pharmacokinetics, therefore improving the bioavailability of drugs in the eyes. Nanomedicine has the characteristics of offering ultra-small size particles, having versatility and target specificity, and have recently been widely investigated for treating DED [206]. Drugs formulated as nanoparticles can enhance the treatment of DED compared to topical treatments. This is a new trend for the future. Positively charged nanoparticles (NPs) with a diameter of 250 nm consisting of EGCG with surface decoration by HA results increased tear volume, reduces *IL1B* and *IL6* gene expression, and restores normal corneal architecture with improving associated clinical signs [207]. PLA-b-Dex-g-PBA NPs (mean diameter = 35.6 ± 7.4) encapsulating up to 12 wt.% of CsA can target the mucous membrane. These demonstrate promising treatment of DED by showing no signs of physical irritation or inflammatory responses after 1 and 12 weeks treatment in an animal model with once a week dosage [208]. Cationized NPs constructed by gelatin and a plasmid coding a modified MUC5AC protein (pMUC5AC) can induce the expression of modified MUC5A and decrease CD4+ T-cell infiltration, and consequently improve the clinical signs [209]. A potential treatment for DED by applying poly(catechin) capped-gold nanoparticles (Au@Poly-CH NPs) with amfenac (F; a nonsteroidal anti-inflammatory drug (NSAID)) (core: 57.51 ± 3.92 nm, shell: 18.52 nm ± 4.37 nm) delivers anti-inflammatory and anti-oxidative treatment simultaneously [210]. Drugs formulated as NPs can enhance the treatment of DED with longterm drug release capacity to reduce dosing frequency ompared to topical treatments. Hence, it is predicted to be a new trend for the future.

In summary, based on the advances in treating DED and improving understanding of its pathophysiology, more treatments were found to be alternate options for patients. In the future, pharmaceutical agents moving toward the market for treating DED are inevitable, and many of them will have new problems or side effects. Regarding the chronic nature of DED, how well these medications will be tolerated when taken over a long time period, and whether their benefit is maintained when taken chronically, remains to be seen [211]. Undoubtedly, as clinical supervisors, we have the responsibility to enaure the efficacy and safety of novel treatments for DED. The literature on NPs for DED treatment is summarized in Table 2.

## 7. Conclusions

Dry eye disease is a complicated ocular disorder with several common subtypes. A comprehensive history and investigation would help ophthalmologists to identify each cause of dry eye and its subsequent management. Traditional medications such as artificial tears, gels and ointments are commonly prescribed for mild to moderate disease. Besides, other treatment modalities such as immune-modulating drugs, topical steroid, antibiotics and bandage contact lenses might be used in more severe cases. The DED epidemiology in Taiwan acquired from the NHIRD reveals that DED rate is around one fourth of the total population related to age, gender, and environmental factors. Many new therapeutic methods such as artificial solutions with anti-inflammatory agents or nanomedicine containing eye drops have been studied. This review summarizes the epidemiology, common subtypes and treatment of dry eye diseases from the clinic to the benchside, in order to clearly understand this disease and effectively manage it in the future.

## Figures and Tables

**Table 1:** Assessments for the diagnosis and evaluation of dry eye disease (DED).

Assessment Tool	Evaluation	Ref.
Corneal fluorescein staining	Corneal and conjunctival vital dye staining (fluorescein/rose Bengal) to identify and track ocular-surface changes at the cellular level.	[5]
Schirmer test	Measurement of tear volume by placing a paper test strip in the lateral third of the lower eyelid after drying the inferior fornix, then measuring the length of the moistened portion of the strip after 5 min.	[9]
Tear osmolarity	Measurement of solutes of tear film (e.g., TearLab™ osmolarity system)	[10]
Tear film stability	Fluorescein tear break-up time widely used to assess tear film stability and reflect different pathophysiologies by break-up pattern.	[11,12]
Tear film interferometry	Assessment of the thickness of the superficial lipid layer that floats upon the tear film and of the fluid layer that covers the anterior surface of contact lenses (to reflect clinical tear dynamics of DED	[13,14]
Meibomian gland grading	Grading of Meibomian gland dysfunction according to clinical features and gland expression	[15]
Inflammation examination	Measurement of matrix metalloproteinase 9 (MMP9) level in the tear film to identify patients with ocular surface inflammation and autoimmune disease (levels >40 ng/mL indicate ocular surface inflammation)	[16,17]
Questionnaires to check patient’s lifestyle or suffering history	Questionnaires assessing patient’s subjective experience of DED in order to get more objective and reproducible data as reference to diagnosis:, model questionnaire can be acquired from: - National Eye Institute Visual Function Questionnaire-25 (NEI-VFQ25) - Ocular Surface Disease Index (OSDI) - Subject Global Assessment scale - Standard Patient Evaluation of Eye Dryness Questionnaire (SPEED) - Canadian Dry Eye Epidemiology Study (CANDEES) - Dry Eye Screening for Dry Eye Epidemiology Projects (DEEP) - Dry Eye Questionnaire (DEQ) - Contact Lens Dry Eye Questionnaire (CLDEQ) - Impact of Dry Eye in Everyday Life (IDEEL) - McCarty Symptom Questionnaire - McMonnies Questionnaire - Ocular Comfort Index (OCI) - Symptom Assessment In Dry Eye (SANDE) - Schein Questionnaire - Texas Eye Research and Technology Center Dry Eye Questionnaire (TERTC-DEQ)	[7,18,19,20]

**Table 2:** New trend for nanomedicine in DED treatment.

Polymer/Material	Drug	Treatment Effects	Ref.
Gelatin	MUC5AC protein (pMUC5AC)	No ocular discomfort and irritation in vivo Normal architecture and morphology Decreases in CD4+ T-cell infiltration Improves associated clinical signs such as tear secretion and fluorescein staining recovered	[191]
Gelatin, hyaluronic acid (HA)	Epigallocatechin gallate (EGCG)	Reduces the *IL1B* and *IL6 gene expression,* Accumulates a lot of nanoparticles in cytoplasm of HCECs and also the ocular surface Displays normal corneal architecture Improves associated clinical signs such as tear secretion and fluorescein staining recovered.	[207]
Gelatin-*g*-Poly(*N*-isopropylacrylamide) (GN)	Epigallocatechin gallate (EGCG)	Sustains the release of EGCG without drug toxicity Prevents further tear evaporation and loss of mucin- secreting goblet cells Reduces ROS, and IL1β and MCP-1 expression Ameliorates corneal epithelial defects	[212]
Poly (d,l-lactide), Dextran and 3-aminophenylboronic acid monohydrate (PLA-b-Dex-g-PBA)	Cyclosporine A (CsA)	Eliminates Inflammatory infiltrates Recovers the ocular surface completely No signs of physical irritation or inflammatory responses Reduces the frequency of administration Increases the retention time on the ocular surface Increases goblet cells	[190]
Poly(d,l-lactide-co-glycolide) (PLGA)	CsA	Sustainable drug release for a long period	[213]
(ethylene glycol)-poly (lactide) polymer (mPEG-PLA)	CsA	Sustainable drug release and concentration for a long period Increases the retention time on the ocular surface Less cytotoxicity than pure CsA	[214]
Methoxy-poly(ethylene glycol), hexyl-substituted poly(lactides) (MPEG-hexPLA)	CsA	Reduces local side effects such as burning and eye pain No cytotoxicity No negative effects on tear production and basal ocular conditions Provides effective and selective drug delivery	[215]
Poly(catechin) capped- gold nanoparticles (Au@Poly-CH NPs)	Amfenac [AF; a nonsteroidal antiinflammatory drug (NSAID)]	Blocks the cyclooxygenase enzymes-induced inflammation and reactive oxygen species (ROS)-induced oxidative stress simultaneously	[192]
Phosphatidylcholine, cholesterol/gellan gum, hydroxypropyl methylcellulose, levocarnitine	Vitamins A and E	No cytotoxicity No discomfort and clinical signs Has high potential to replenish tear film lipids, restore the tear film and protect corneal epithelium	[216]
